# Evaluation of the application value of ultrasound in the diagnosis of community-acquired pneumonia in children: a preliminary study based on large-scale outpatient cases

**DOI:** 10.3389/fped.2025.1582647

**Published:** 2025-04-30

**Authors:** Tingting Qin, Zengpo Huang, Qiuhui Lan, Yijun Nie, Guofei Luo, Lixue Cheng, Linglin Li, Zhixuan Tang, Zhao Ma

**Affiliations:** ^1^ Department of Pediatrics, Liuzhou People's Hospital, Liuzhou, China; ^2^ Department of Ultrasound Medicine, Liuzhou People's Hospital, Liuzhou, China

**Keywords:** children, community-acquired pneumonia, diagnostic value, lung ultrasound (LUS), outpatient

## Abstract

**Objective:**

To evaluate the diagnostic value of lung ultrasound (LUS) for childhood community-acquired pneumonia (CAP).

**Methods:**

A case-control study was conducted among pediatric outpatients with suspected CAP who underwent LUS examination. Baseline data such as clinical manifestations were collected. Diagnostic performance was assessed using Pearson Chi-square tests, with CT scans as the gold standard. Specificity, sensitivity, positive predictive value (PPV), and negative predictive value (NPV) of LUS were analyzed.

**Results:**

The study included 246 patients who underwent both LUS and CT (observation group) and 111 controls who received concurrent chest x-ray and CT within one week. LUS demonstrated significantly higher sensitivity (91.30% vs. 75.00%, *P* < 0.001) than chest x-ray group, while specificity, PPV, and NPV showed no statistically significant (*P* > 0.05).

**Conclusion:**

LUS exhibits high concordance with CT in pediatric CAP diagnosis, demonstrating excellent screening and diagnostic value for childhood CAP.

## 1 Introduction

Community-acquired pneumonia (CAP) is a common respiratory disease in children where early and accurate diagnosis is crucial for reducing complications and mortality (1). Currently, CT scans provide detailed pulmonary imaging and are more accurate for diagnosing complex or severe CAP (2), but concerns about radiation exposure and cost restricted their application in pediatric populations (3, 4). In recent years, lung ultrasound (LUS) has gained attention in CAP diagnosis due to its radiation-free, convenient, and bedside operation (5–7). While numerous studies have analyzed the sensitivity and specificity of LUS for diagnosing pneumonia in adults, research in pediatric outpatient settings remains limited (8–10). This study aims to investigate the diagnostic value of LUS for childhood CAP through a large-scale analysis of pediatric outpatient cases.

## 2 Methods

### 2.1 Data collection and grouping

This is a retrospective case-control study. Clinical diagnosis and treatment were performed by pediatricians. All cases were managed according to the *Diagnosis and Treatment Guidelines for Pediatric Community-Acquired Pneumonia (2019 Edition)* issued by the National Health Commission of China. We enrolled children aged ≤14 years with suspected CAP who visited the pediatric outpatient department of Liuzhou People's Hospital between January 1, 2021, and September 7, 2024. Clinical data were extracted from medical record, including gender, age, underlying diseases, symptoms (fever, cough, chest pain, asthma), pulmonary signs (coarse breath sounds, wheezing sound, fixed crackles), and imaging findings. Patients with primary pulmonary/respiratory diseases or non-Chinese nationality were excluded. Data collection was implemented by Tingting Qin, Zengpo Huang and Qiuhui Lan. And standardization was implemented by Linglin Li and Zhixuan Tang. Zhao Ma was responsible for quality control.

**Figure 1:**
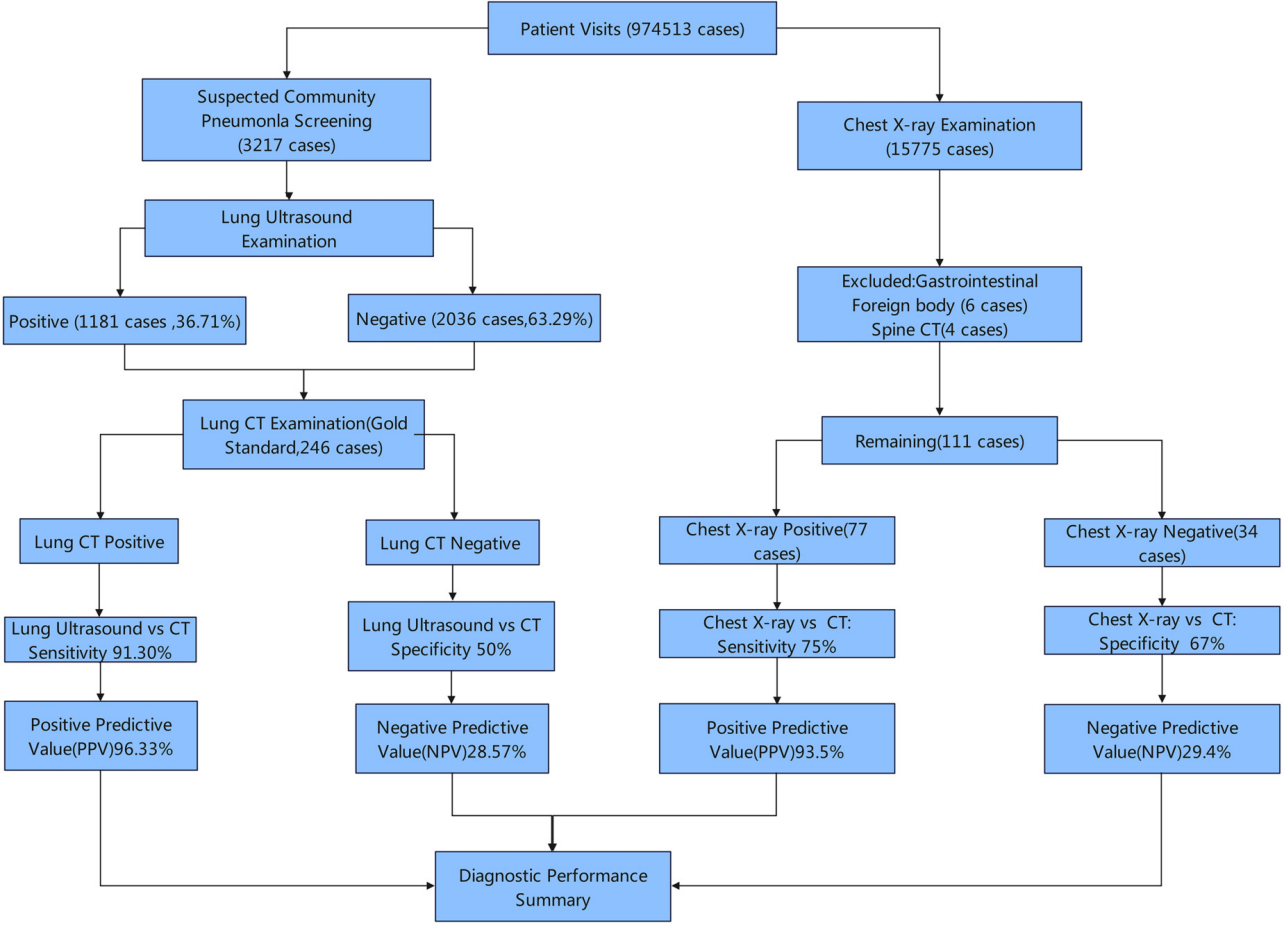
Study design.

Cases that underwent both LUS and CT scans were included in the observation group, while cases that underwent both x-ray and CT scan within one week included in the control group. Using CT scans as the gold standard, the specificity, sensitivity, positive predictive value (PPV), and negative predictive value (NPV) of LUS in diagnosis of CAP were analyzed. For more detail, the study design is presented in Figure 1.

**Figure 2:**
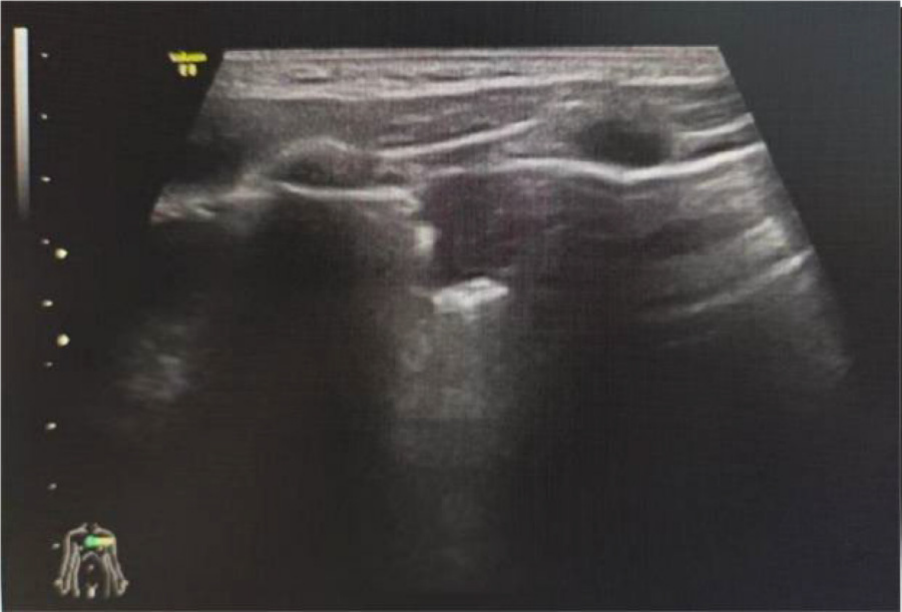
Fragmentation sign (patient 1).

**Figure 3:**
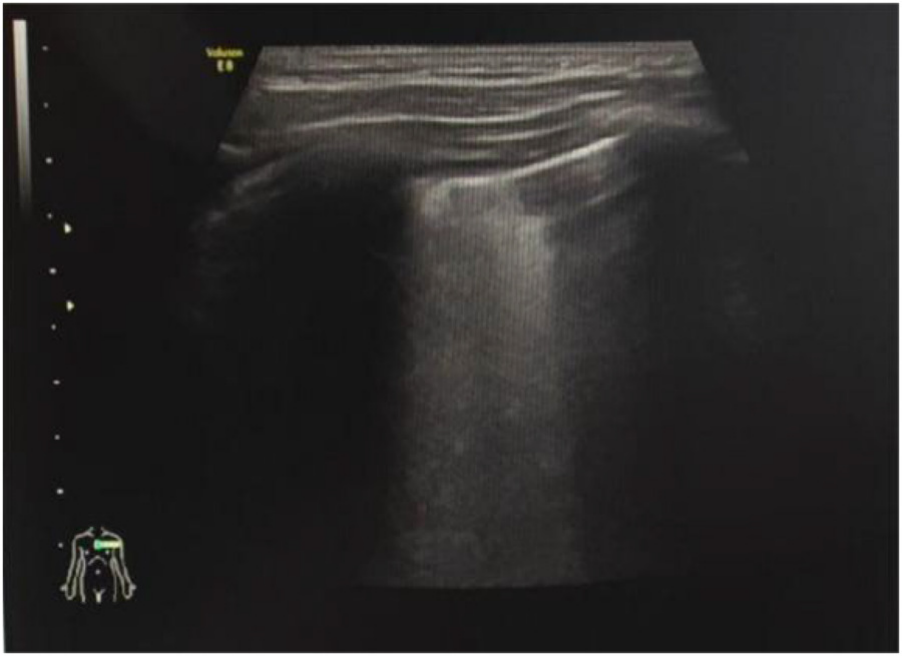
B-line (patient 2).

The Voluson E8 ultrasound system from GE Healthcare Austria GmbH was used in this study. LUS was performed by the pediatric specialized team in the department of ultrasound medicine, with Lixue Cheng responsible for quality control. The presence of A-lines and sliding sign on LUS was defined as negative. In the observation group, different ultrasound images was subdivided into Observation Group 1–5:

Observation Group 1: consolidation or lung fragmentation signs (Figure 2).
Observation Group 2: diffuse B-lines (Figure 3).
Observation Group 3: multiple B-lines (defined as ≥3 B-lines in any intercostal space).
Observation Group 4: few B-lines (defined as <3 B-lines in any intercostal space).
Observation Group 5: hydrothorax.

Chest x-ray and CT scan were implemented and diagnosed by the medical imaging center of Liuzhou People's Hospital.

This study has been approved by the Medical Ethics Committee of Liuzhou People's Hospital (No. KY-2023-075).

### 2.2 Statistical analysis

Data analysis was performed using IBM SPSS 24.0 software by Yijun Nie and Guofei Luo. Differences between the two groups were compared using Pearson Chi-square test or Fisher's exact test (*α* = 0.05).

## 3 Results

A total of 3,217 children with suspected CAP underwent LUS examination, with 1,181 cases (36.71%) testing positive and 2,036 cases (63.29%) negative. Among them, 246 patients who received concurrent LUS and chest CT scan within one week were enrolled in observation group, while 111 age-matched subjects undergoing the same examinations were selected as controls. Demographic details are presented in Table 1.

**Table 1:** Demographic characteristics of children suspected of community-acquired pneumonia.

Demographic characteristics [*n* (%)]	Ultrasound positive group [*n* = 218 (89)]	Ultrasound negative group [*n* = 28 (11)]	X*-*ray Positive Group [*n* = 77 (69)]	X*-*ray negative group [*n* = 34 (31)]	*P*-value^a^
Gender					0.725
Male	104 (48)	13 (46)	34 (44)	19 (56)	
Female	114 (52)	15 (54)	43 (56)	15 (44)	
Age Group					<0.001
Under 1 month	2 (1)	0	0	0	
1 month–1 year	61 (28)	8 (29)	2	1	
Over 1 year	155 (71)	20 (71)	75	33	
Systemic symptoms					0.054
Fever	159 (73)	19 (68)			
Cough	178 (82)	28 (100)			
Chest pain	22 (10)	1 (4)			
Asthma	96 (44)	4 (14)			
Signs					<0.001
Only coarse breath sounds	102 (57)	20 (76)			
Wheezing sound	41 (21)	1 (15)			
Fixed wet rales	81 (22)	0			
Ultrasound imaging characteristics			–	–	–
Consolidation	113 (52)	0			
Diffuse B-lines	7 (3)	0			
Multiple B-lines	20 (9)	0			
Few B-lines	70 (32)	0			
Hydrothorax	8 (4)	0			
A-line with sliding sign.	0	28 (100)			

^a^ Pearson's Chi-Square Test.

Using chest CT scans as the gold standard, the diagnostic performance of LUS was evaluated as follows: sensitivity of 91.30%, specificity of 50%, PPV of 96.33% and NPV of 28.57%. The sensitivity of LUS was significantly higher than that of the chest x-ray group (*P* < 0.001), while the specificity, PPV and NPV showed no significantly difference (all *P* > 0.05), as illustrated in Table 2.

**Table 2:** Comparison of the diagnostic performance of children community-acquired pneumonia between pulmonary ultrasound and chest x-ray.

Group	TP	FP	FN	TN	Sensitivity (0.95CI)	Specificity (0.95CI)	PPV	NPV	Kappa
Ultrasound	210	8	20	8	91.30% (87.67%–94.94%)	50% (25.50%–74.50%)	96.33%	28.57%	0.31
Chest x-ray	72	5	24	10	75.0% (66.34%–83.66%)	66.67% (42.81%–90.52%)	93.51%	29.41%	0.27
*P*-value^a^					<0.001	0.473	0.546	0.726	

TP, true positive; FP, false positive; FN, false negative; TN, true negative; PPV, positive predictive value; NPV, negative predictive value.

^a^ Pearson's Chi-Square Test.

Respectively, 113, 7, 20, 65 and 8 cases were subdivided into observation group 1–5 based on their different LUS images. The sensitivity (*P* < 0.001) and specificity (*P* < 0.05) showed significantly different than that of the chest x-ray group, while the PPV and NPV were no significantly difference, as shown in Table 3.

**Table 3:** Comparison of the diagnostic performance of children community-acquired pneumonia among different pulmonary ultrasound images and chest x-ray.

Group	TP	FP	FN	TN	Sensitivity (0.95CI)	Specificity (0.95CI)	PPV (0.95CI)	NPV (0.95CI)
Observation group 1	110	3	20	8	84.62% (78.26%–90.98%)	72.73% (46.50%–98.96%)	97.35% (94.34%, 100%)	28.57% (11.74%–45.40%)
Observation group 2	7	0	20	8	25.93% (9.47%–42.39%)	100% (1)	100% (1)	28.57% (11.74%–45.40%)
Observation group 3	20	0	20	8	50% (34.51%–65.49%)	100% (1)	100% (1)	28.57% (11.74%–45.40%)
Observation group 4	65	5	20	8	76.47% (67.34%–85.60%)	61.54% (35.21%–87.87%)	92.86% (86.79%, 99.33%)	28.57% (11.74%–45.40%)
Observation group 5	8	0	20	8	28.57% (11.74%–45.40%)	100% (1)	100% (1)	28.57% (11.74%–45.40%)
Control group	72	5	24	10	75% (66.34%–83.66%)	66.67% (42.91%–90.43%)	93.51% (88.00%, 99.02%)	29.4% (13.91%–44.91%)
*P*-value^a^					<0.001	<0.05	>0.05	>0.05

TP, true positive; FP, false positive; FN, false negative; TN, true negative; PPV, positive predictive value; NPV, negative predictive value.

^a^ Pearson's Chi-Square Test, and Fisher's Exact Probability.

## 4 Discussion

LUS has been widely applied in intensive care units, demonstrating significantly enhanced sensitivity for critically ill patients (11). However, most studies on CAP diagnosis, particularly outpatient CAP, have primarily focused on adults populations. Research indicates that LUS achieves a sensitivity of 93.4% and a specificity of 97.7% in diagnosing CAP in adults (12). Furthermore, studies have explored its role in emergency department settings, revealing both improved diagnostic accuracy and reduced diagnostic uncertainty (13). Additional dedicated studies have evaluated the diagnostic performance (sensitivity and specificity) of LUS for pneumonia in adults (14).

The sensitivity of LUS for diagnosing CAP in outpatient children was 91.30%, capable of detecting 91.30% of true positive cases with a low missed diagnosis rate. The diagnostic performance of outpatient lung ultrasound for childhood CAP was significantly higher than that of chest x-ray, with a statistically significant superiority (*P* < 0.001). However, the specificity of LUS was 50%, relatively low, suggesting a possibility of false positives. Therefore, if childhood CAP is high suspected clinically but LUS imaging is negative, further chest CT scans are recommended for evaluation. The PPV of LUS was 96.33%, meaning 96.33% of positive ultrasound results were true positives. This highlights its excellent performance in diagnosis and provides an advantage over chest x-ray, as a positive ultrasound can essentially confirm the diagnosis of childhood CAP. On the other hand, the NPV is 28.57%, indicating that only 28.57% of negative results were true negatives, which warrants caution in interpreting negative ultrasound findings. Overall, LUS exhibits high sensitivity and PPV in identifying childhood CAP, making it a more suitable initial screening tool than chest x-ray. Despite its lower specificity, negative ultrasound results should be comprehensively evaluated in conjunction with other diagnostic methods, such as CT scans. In the consolidation subgroup, it demonstrated a sensitivity of 84.62%, specificity of 72.73%, and a PPV of 97.35%. The consolidation sign, with their high sensitivity and specificity, serve as critical evidence for diagnosing childhood CAP via LUS.

Three cases showed lung consolidation on ultrasound but no abnormalities on chest CT in this study. Smaller consolidations may be less evident on CT imaging but can be visualized by LUS. Therefore, detection of small consolidations via LUS may suggest the early stage of pneumonia, which may warrant further investigation.

The limitations of this study include that only a small subset of cases underwent CT verification after pulmonary ultrasound results, leading to potential sample representativeness bias. Future multicenter, large-sample studies of ultrasound across diverse populations and healthcare settings may provide more strong evidence.

## 5 Conclusion

LUS demonstrates higher sensitivity than chest x-ray, suggesting superior diagnostic performance for childhood CAP, particularly in identifying lung consolidation and minor B-line signs. Considering its convenience, it may serve as an effective screening and diagnostic method in children CAP. For those who got positive symptoms and negative pulmonary ultrasound imaging, further chest CT scans are recommended to provide more comprehensive and accurate evidence for suspected cases.

## Data Availability

The raw data supporting the conclusions of this article will be made available by the authors, without undue reservation.

## References

[1] Zhong H Dong X. Analysis of clinical characteristics and risk factors of severe adenovirus pneumonia in children. Front Pediatr. (2021) 9:566797. 10.3389/fped.2021.566797 34712627 PMC8546294

[2] National Health Commission of the People’s Republic of China. Guidelines for the diagnosis and treatment of community-acquired pneumonia in children. Chin J Clin Infect Dis. (2019) 12(1):6–13. 10.3760/cma.j.issn.1674-2397.2019.01.002

[3] Mandell LA Wunderink RG Anzueto A Bartlett JG Campbell GD Dean NC Infectious Diseases Society of America/American Thoracic Society consensus guidelines on the management of community-acquired pneumonia in adults. Clin Infect Dis. (2007) 44(Suppl 2):S27–72. 10.1086/511159 17278083 PMC7107997

[4] Chavez MA Shams N Ellington LE Naithani N Gilman RH Steinhoff MC Lung ultrasound for the diagnosis of pneumonia in adults: a systematic review and meta-analysis. Respir Res. (2014) 15(1):50. 10.1186/1465-9921-15-50. 24758612 PMC4005846

[5] Lichtenstein D Mézière G Biderman P Gepner A Barré O. The comet-tail artifact. An ultrasound sign of alveolar-interstitial syndrome. Am J Respir Crit Care Med. (1997) 156(5):1640–6. 10.1164/ajrccm.156.5.96-07096 9372688

[6] Lichtenstein D. Lung ultrasound in the critically ill. Curr Opin Crit Care. (2014) 20(3):315–22. 10.1097/MCC.0000000000000096 24758984

[7] Vaughn VM Dickson RP Horowitz JK Flanders SA. Community-acquired pneumonia: a review. JAMA. (2024) 332(15):1282–95. 10.1001/jama.2024.14796 39283629

[8] Orso D Guglielmo N Copetti R. Lung ultrasound in diagnosing pneumonia in the emergency department: a systematic review and meta-analysis. Eur J Emerg Med. (2018) 25(5):312–21. 10.1097/MEJ.0000000000000517 29189351

[9] Reissig A Gramegna A Aliberti S. The role of lung ultrasound in the diagnosis and follow-up of community-acquired pneumonia. Eur J Intern Med. (2012) 23(5):391–7. 10.1016/j.ejim.2012.01.003 22726366

[10] Musolino AM Di Sarno L Buonsenso D Murciano M Chiaretti A Boccuzzi E Use of POCUS for the assessment of dehydration in pediatric patients-a narrative review. Eur J Pediatr. (2024) 183(3):1091–105. 10.1007/s00431-023-05394-2 38133810

[11] Staub LJ Mazzali Biscaro RR Kaszubowski E Maurici R. Lung ultrasound for the emergency diagnosis of pneumonia, acute heart failure, and exacerbations of chronic obstructive pulmonary disease/asthma in adults: a systematic review and meta-analysis. J Emerg Med. (2019) 56(1):53–69. 10.1016/j.jemermed.2018.09.009 30314929

[12] Boccatonda A Cocco G D'Ardes D Delli Pizzi A Vidili G De Molo C Infectious pneumonia and lung ultrasound. A Review. J Clin Med. (2023) 12(4):1402. 10.3390/jcm12041402 36835938 PMC9964129

[13] Javaudin F Marjanovic N de Carvalho H Gaborit B Le Bastard Q Boucher E Contribution of lung ultrasound in diagnosis of community-acquired pneumonia in the emergency department: a prospective multicentre study. BMJ Open. (2021) 11(9):e046849. 10.1136/bmjopen-2020-046849 34561254 PMC8475146

[14] Long L Zhao HT Zhang ZY Wang GY Zhao HL. Lung ultrasound for the diagnosis of pneumonia in adults: a meta-analysis. Medicine (Baltimore). (2017) 96(3):e5713. 10.1097/MD.0000000000005713 28099332 PMC5279077

